# Supplementary material for: The Effectiveness of Different Doses of Iron Supplementation and the Prenatal Determinants of Maternal Iron Status in Pregnant Spanish Women: ECLIPSES Study
Source: Nutrients. 2019 Oct 10;11(10):2418. doi: 10.3390/nu11102418 (PMC6835785; doi:10.3390/nu11102418)
Supplement: Supplementary file 1 [file nutrients-11-02418-s001.zip › Tablas 3S-4S.docx]

| **Table 3S. Effect of the intervention with iron supplementation (40 or 80 mg/day) through pregnancy on hemoglobin and serum ferritin levels and on the risk of ID, anemia, IDA and hemoconcentration at third trimester in women from *Stratum* 1 (by protocol).** | | | | |
| --- | --- | --- | --- | --- |
| **Hemoglobin levels** |  |  |  |  |
| **Independent variables** | **B** | **SE** | **p** | **Model** |
| ^a^ Intervention (0:80 mg/d, 1:40mg/d) | -0,17 | 0,85 | 0,839 | R2c.100=-0,3; F321,1=0,04; p=0,839 |
| ^b^ Intervention (0:80 mg/d, 1:40mg/d) | -0,09 | 0,92 | 0,920 | R2c.100 = 0,5; F258,11=1,11; p=0,356 |
| Serum ferritin at 12^th^ week of pregnancy | 1,65 | 0,70 | 0,018 |  |
| **Serum ferritin levels** |  |  |  |  |
| **Independent variables** | **B** | **SE** | **p** | **Model** |
| ^a^ Intervention (0:80 mg/d, 1:40mg/d) | -0,10 | 0,06 | 0,093 | R2c.100=0,4; F350,1=2,84; p=0,093 |
| ^c^ Intervention (0:80 mg/d, 1:40mg/d) | -0,14 | 0,06 | 0,010 | R2c.100 = 45,1; F342,11=26,56; p<0,001 |
| Serum ferritin at 12^th^ week of pregnancy | 0,62 | 0,04 | <0,001 |  |
| Maternal age (0:25-34 years, 1:<25 years) | -0,01 | 0,09 | 0,975 |  |
| Maternal age (0:25-34 years, 1:≥35 years) | 0,22 | 0,07 | 0,001 |  |
| **Iron deficiency (0:no, 1:yes)** |  |  |  |  |
| **Independent variables** | **OR** | **95% CI** | **p** | **Model** |
| ^a^ Intervention (0:80 mg/d, 1:40mg/d) | 1,71 | 1,10–2,63 | 0,016 | R2 Nagelkerke.100 = 2,3; X2_1,1_ = 5,85; p=0,016 |
| ^c^ Intervention (0:80 mg/d, 1:40mg/d) | 1,90 | 1,10–3,28 | 0,022 | R2 Nagelkerke.100 = 25,4; X2_11,1_ = 55,64; p>0,001 |
| Serum ferritin at 12^th^ week of pregnancy | 0,26 | 0,16–0,42 | <0,001 |  |
| **Anemia (0:no, 1:yes)** |  |  |  |  |
| **Independent variables** | **OR** | **95% CI** | **p** | **Model** |
| ^a^ Intervention (0:80 mg/d, 1:40mg/d) | 1,19 | 0,56–2,53 | 0,649 | R2 Nagelkerke.100 = 0,1; X2_1,1_ = 0,21; p=0,649 |
| ^b^ Intervention (0:80 mg/d, 1:40mg/d) | 2,03 | 0,79–5,17 | 0,140 | R2 Nagelkerke.100 = 12,8; X2_11,1_ = 15,39; p=0,165 |
| Serum ferritin at 12^th^ week of pregnancy | 0,41 | 0,21–0,80 | 0,009 |  |
| **Iron-deficiency anemia (0:no, 1:yes)** |  |  |  |  |
| **Independent variables** | **OR** | **95% CI** | **p** | **Model** |
| ^a^ Intervention (0:80 mg/d, 1:40mg/d) | 0,94 | 0,40–2,19 | 0,877 | R2 Nagelkerke.100 = 0,0; X2_1,1_ = 0,02; p=0,877 |
| ^b^ Intervention (0:80 mg/d, 1:40mg/d) | 1,46 | 0,53–4,02 | 0,460 | R2 Nagelkerke.100 = 17,0; X2_11,1_ = 18,64; p=0,068 |
| Serum ferritin at 12^th^ week of pregnancy | 0,27 | 0,13–0,57 | <0,001 |  |
| **Hemoconcentration (0:no, 1:yes)** |  |  |  |  |
| **Independent variables** | **OR** | **95% CI** | **p** | **Model** |
| ^a^ Intervention (0:80 mg/d, 1:40mg/d) | 1,22 | 0,54–2,72 | 0,635 | R2 Nagelkerke.100 = 0,1; X2_1,1_ = 0,23; p=0,635 |
| ^b^ Intervention (0:80 mg/d, 1:40mg/d) | 1,47 | 0,53–4,06 | 0,462 | R2 Nagelkerke.100 = 16,8; X2_11,1_ = 17,89; p=0,084 |
| Genotype HFE (0:WT/WT, 1: carrier of H63D) | 3,23 | 1,13–9,20 | 0,028 |  |
| Genotype HFE (0:WT/WT, 1: C282Y/WT) | 1,74 | 0,19–16,21 | 0,627 |  |
| Parity (0:no, 1:yes) | 0,27 | 0,09–0,78 | 0,016 |  |
| ^a^ Crude model | | | | |
| ^b^ Adjusted for: dose of iron supplementation, maternal age, use of hormonal contraceptives, pre-pregnancy maternal body mass index, weight gain in pregnancy, genotypes of HFE gene, maternal ethnic origin, hemoglobin at 12^th^ week, serum ferritin at 12^th^ week, C-reactive protein at 12^th^ week, socioeconomic status, weekly mean of METS at 12^th^ week, smoking habit, alcohol intake, pregnancy planning, parity, mean calories intake during pregnancy, MED score | | | | |
| ^c^ Adjusted for: model b except for hemoglobin at 12^th^ week | | | | |

| **Table 4S. Effect of the intervention with iron supplementation (40 or 20 mg/day) through pregnancy on hemoglobin and serum ferritin levels and on the risk of ID, anemia, IDA and hemoconcentration at third trimester in women from *Stratum* 2 (by protocol).** | | | | |
| --- | --- | --- | --- | --- |
| **Hemoglobin levels** |  |  |  |  |
| **Independent variables** | **B** | **SE** | **p** | **Model** |
| ^a^ Intervention (0:40 mg/d, 1:20mg/d) | -2,16 | 1,43 | 0,133 | R2c.100=0,7; F178,1=2,28; p=0,133 |
| ^b^ Intervention (0:40 mg/d, 1:20mg/d) | -2,61 | 1,47 | 0,079 | R2c.100=11,2; F145,11=2,67; p=0,004 |
| Genotype HFE (0:WT/WT, 1: carrier of H63D) | 3,79 | 1,74 | 0,032 |  |
| Genotype HFE (0:WT/WT, 1: C282Y/WT) | 1,19 | 3,71 | 0,748 |  |
| Hemoglobin at 12^th^ week of pregnancy | 0,71 | 0,16 | <0,001 |  |
| **Serum ferritin levels** |  |  |  |  |
| **Independent variables** | **B** | **SE** | **p** | **Model** |
| ^a^ Intervention (0:40 mg/d, 1:20mg/d) | 0,03 | 0,07 | 0,670 | R2c.100=-0,3; F179,1=0,18; p=0,670 |
| ^c^ Intervention (0:40 mg/d, 1:20mg/d) | 0,03 | 0,08 | 0,672 | R2c.100 = 25,4; F145,13=6,14; p<0,001 |
| Maternal age (0:25-34 years, 1:<25 years) | -0,26 | 0,11 | 0,020 |  |
| Maternal age (0:25-34 years, 1:≥35 years) | 0,11 | 0,10 | 0,248 |  |
| Serum ferritin at 12^th^ week of pregnancy | 0,42 | 0,06 | <0,001 |  |
| Smoking habit (0:no, 1:yes) | 0,22 | 0,10 | 0,023 |  |
| Alcohol intake (0:no, 1:yes) | -1,15 | 0,54 | 0,034 |  |
| **Low iron stores (0:no, 1:yes)** |  |  |  |  |
| **Independent variables** | **OR** | **95% CI** | **p** | **Model** |
| ^a^ Intervention (0:40 mg/d, 1:20mg/d) | 1,18 | 0,63–2,18 | 0,614 | R2 Nagelkerke.100 = 0,2; X2_1,1_ = 0,26; p=0,614 |
| ^c^  Intervention (0:40 mg/d, 1:20mg/d) | 1,36 | 0,64–2,86 | 0,423 | R2 Nagelkerke.100 = 22,3; X2_11,1_ = 26,70; p=0,005 |
| Serum ferritin at 12^th^ week of pregnancy | 0,35 | 0,18–0,68 | 0,002 |  |
| **Anemia (0:no, 1:yes)** |  |  |  |  |
| **Independent variables** | **OR** | **95% CI** | **p** | **Model** |
| ^a^ Intervention (0:40 mg/d, 1:20mg/d) | 1,47 | 0,55–3,92 | 0,441 | R2 Nagelkerke.100 = 0,7; X2_1,1_ = 0,60; p=0,440 |
| ^b^ Intervention (0:40 mg/d, 1:20mg/d) | 1,30 | 0,31–5,48 | 0,720 | R2 Nagelkerke.100 = 39,5; X2_13,1_ = 28,66; p=0,007 |
| Serum ferritin at 12^th^ week of pregnancy | 0,21 | 0,07–0,63 | 0,006 |  |
| SES (0:low; 1:middle + high) | 0,09 | 0,02–0,50 | 0,006 |  |
| **Iron-deficiency anemia (0:no, 1:yes)** |  |  |  |  |
| **Independent variables** | **OR** | **95% CI** | **p** | **Model** |
| ^a^ Intervention (0:40 mg/d, 1:20mg/d) | 1,70 | 0,62–4,68 | 0,306 | R2 Nagelkerke.100 = 1,3; X2_1,1_ = 1,07; p=0,302 |
| ^b^ Intervention (0:40 mg/d, 1:20mg/d) | 1,30 | 0,31–5,48 | 0,720 | R2 Nagelkerke.100 = 39,5; X2_13,1_ = 28,66; p=0,007 |
| Serum ferritin at 12^th^ week of pregnancy | 0,21 | 0,07–0,63 | 0,006 |  |
| SES (0:low; 1:middle + high) | 0,09 | 0,02–0,50 | 0,006 |  |
| **Hemoconcentration (0:no, 1:yes)** |  |  |  |  |
| **Independent variables** | **OR** | **95% CI** | **p** | **Model** |
| ^a^ Intervention (0:40 mg/d, 1:20mg/d) | 0,47 | 0,21–1,04 | 0,062 | R2 Nagelkerke.100 = 3,3; X2_1,1_ = 3,67; p=0,056 |
| ^b^ Intervention (0:40 mg/d, 1:20mg/d) | 0,31 | 0,10–0,91 | 0,034 | R2 Nagelkerke.100 = 28,1; X2_11,1_ = 27,38; p=0,004 |
| Hemoglobin at 12^th^ week of pregnancy | 1,20 | 1,08–1,33 | 0,001 |  |
| Genotype HFE (0:WT/WT, 1: carrier of H63D) | 3,04 | 1,08-8,57 | 0,036 |  |
| Genotype HFE (0:WT/WT, 1: C282Y/WT) | 0,00 | . | 0,999 |  |
| ^a^ Crude model | | | | |
| ^b^ Adjusted for: dose of iron supplementation, maternal age, use of hormonal contraceptives, pre-pregnancy maternal body mass index, weight gain in pregnancy, genotypes of HFE gene, maternal ethnic origin, hemoglobin at 12^th^ week, serum ferritin at 12^th^ week, C-reactive protein at 12^th^ week, socioeconomic status, weekly mean of METS at 12^th^ week, smoking habit, alcohol intake, pregnancy planning, parity, mean calories intake during pregnancy, MED score | | | | |
| ^c^ Adjusted for: model b except for hemoglobin at 12^th^ week | | | | |
