# Supplementary material for: The Effectiveness of Different Doses of Iron Supplementation and the Prenatal Determinants of Maternal Iron Status in Pregnant Spanish Women: ECLIPSES Study
Source: Nutrients. 2019 Oct 10;11(10):2418. doi: 10.3390/nu11102418 (PMC6835785; doi:10.3390/nu11102418)
Supplement: Supplementary file 1 [file nutrients-11-02418-s001.zip › Table S1.docx]

| **Table 1S. Baseline characteristics of the population lost trhought the study** | | | | | |
| --- | --- | --- | --- | --- | --- |
|  | ***Stratum* 1 (n = 175)** | | ***Stratum* 2 (n = 82)** | | **p** |
|  | **Mean** | **SD** | **Mean** | **SD** |  |
| Age, years | 29,85 | 5,12 | 29,70 | 5,10 | 0,826 |
| Weight, g | 64,48 | 11,43 | 68,10 | 15,49 | 0,061 |
| Pre-pregnancy BMI, Kg/m^2^ | 24,59 | 4,04 | 26,19 | 5,55 | 0,021 |
| Weight gain through pregnancy, g | 11,75 | 11,39 | 8,15 | 15,49 | 0,062 |
|  | **% (n)** | | **% (n)** | |  |
| Smoking habit | 21,1 (37) | | 28,0 (23) | | 0,223 |
| Parity | 68,4 (119) | | 59,8 (49) | | 0,175 |
| Pregnancy planning | 78,9 (138) | | 79,8 (63) | | 0,714 |
| Use of hormonal contraceptives | 14,9 (26) | | 22,0 (18) | | 0,159 |
| Pre-pregnancy BMI |  | |  | |  |
| *Underweight* | 1,7 (3) | | 3,7 (3) | | 0,336 |
| *Normal weight* | 60,6 (106) | | 46,3 (38) | | 0,032 |
| *Overweight* | 26,3 (46) | | 28,0 (23) | | 0,766 |
| *Obesity* | 11,4 (20) | | 22,0 (18) | | 0,027 |
| HFE gene mutation | 34,5 (38) | | 44,8 (26) | | 0,192 |
| HFE genotype |  | |  | |  |
| *WT/WT* | 65,5 (72) | | 55,2 (32) | | 0,192 |
| *C282Y/WT* | 4,6 (5) | | 0,0 (0) | | 0,349 |
| *Carrier of H63D mutation* | 28,7 (31) | | 43,9 (25) | | 0,051 |
| *Carrier of S65C mutation* | 1,8 (2) | | 1,7 (1) | | 0,965 |
| Familiar socioeconomic status |  | |  | |  |
| *Low* | 19,4 (34) | | 19,5 (16) | | 0,987 |
| *Middle* | 65,7 (115) | | 68,3 (56) | | 0,683 |
| *High* | 14,9 (26) | | 12,2 (10) | | 0,567 |
| Maternal ethnic origin |  | |  | |  |
| *Caucasian* | 84,9 (124) | | 84,3 (59) | | 0,902 |
| *Asian* | 0,0 (0) | | 0,0 (0) | | . |
| *Arab* | 10,3 (15) | | 11,4 (8) | | 0,797 |
| *Black* | 0,7 (1) | | 0,0 (0) | | 0,593 |
| *Latin American* | 4,1 (6) | | 4,3 (3) | | 0,952 |
| Adherence to Mediterranean diet |  | |  | |  |
| *Low***–***Middle* | 69,7 (122) | | 74,4 (61) | | 0,44 |
| *High* | 30,3 (53) | | 25,6 (21) | | 0,44 |
| BMI: body mass index; WT: wild type | | | | | |
| * Serum ferritin is expressed in median and interquartile range | | | | | |
| Sample size HFE genotype = 168; sample size maternal ethnic origin = 216 | | | | | |
