# Supplementary material for: The Effectiveness of Different Doses of Iron Supplementation and the Prenatal Determinants of Maternal Iron Status in Pregnant Spanish Women: ECLIPSES Study
Source: Nutrients. 2019 Oct 10;11(10):2418. doi: 10.3390/nu11102418 (PMC6835785; doi:10.3390/nu11102418)
Supplement: Supplementary file 1 [file nutrients-11-02418-s001.zip › Table S2.docx]

| **Table 2S. Biochemical characteristics of participants at 36^th^ week of gestation according to dose of supplementation (by protocol).** | | | | | | |
| --- | --- | --- | --- | --- | --- | --- |
|  | ***Stratum* 1** | | | ***Stratum* 2** | | |
|  | **80 g/d** | **40 g/d** | **p** | **40 g/d** | **20 g/d** | **p** |
| ***12^th^ week*** | | | | | | |
| Hemoglobin (g/L) | 123,87 (4,92) | 123,97 (4,49) | 0,826 | 135,77 (4,63) | 136,71 (4,43) | 0,101 |
| Serum ferritin (µg/L) | 35,70 (21,80) | 37,20 (22,85) | 0,927 | 42,41 (27,00) | 42,40 (32,10) | 0,811 |
| Mean corpuscular volume (fL) | 87,27 (6,46) | 87,59 (6,65) | 0,591 | 88,50 (3,46) | 88,59 (3,77) | 0,842 |
| C–reactive protein (mg/L) | 0,73 (0,61) | 0,73 (0,70) | 0,955 | 0,71 (0,55) | 0,71 (0,53) | 0,932 |
| Iron deficiency (%) | 12,7 (30) | 10,9 (25) | 0,549 | 14,1 (18) | 12,6 (16) | 0,731 |
| ***36^th^ week*** | | | | | | |
| Hemoglobin (g/L) | 118,40 (7,32) | 118,23 (7,89) | 0,839 | 123,20 (10,17) | 121,04 (8,85) | 0,133 |
| Serum ferritin (µg/L) | 17,19 (11,10) | 15,00 (9,75) | 0,035 | 11,00 (7,00) | 11,00 (6,60) | 0,720 |
| Mean corpuscular volume (fL) | 89,33 (7,25) | 88,08 (12,83) | 0,264 | 90,27 (4,27) | 89,62 (4,16) | 0,288 |
| C–reactive protein (mg/L) | 0,78 (0,76) | 0,73 (0,66) | 0,509 | 0,70 (0,69) | 0,75 (0,56) | 0,603 |
| Iron deficiency (%) | 36,7 (62) | 49,7 (84) | 0,016 | 66,7 (66) | 70,1 (61) | 0,614 |
| Iron deficiency anemia (%) | 7,4 (12) | 6,9 (11) | 0,877 | 7,4 (7) | 11,9 (10) | 0,302 |
| Anemia (%) | 8,6 (14) | 10,1 (16) | 0,649 | 8,4 (8) | 11,9 (10) | 0,439 |
| Hemoconcentration (%) | 7,4 (12) | 8,8 (14) | 0,635 | 24,2 (23) | 13,1 (11) | 0,058 |
| Continuous variables were epressed in mean (SD), except for serum ferritin that was expressed in median (interquartile range) | | | | | | |
| Categorical variables were expressed in % (n) | | | | | | |
